# Molecular implications of MUC5AC-CD44 axis in colorectal cancer progression and chemoresistance

**DOI:** 10.1186/s12943-020-01156-y

**Published:** 2020-02-25

**Authors:** Ramesh Pothuraju, Satyanarayana Rachagani, Shiv Ram Krishn, Sanjib Chaudhary, Rama Krishna Nimmakayala, Jawed A. Siddiqui, Koelina Ganguly, Imayavaramban Lakshmanan, Jesse L. Cox, Kavita Mallya, Sukhwinder Kaur, Surinder K. Batra

**Affiliations:** 1 Department of Biochemistry and Molecular Biology, University of Nebraska Medical Center, Omaha, NE USA; 2 Department of Pathology and Microbiology, University of Nebraska Medical Center, Omaha, NE USA; 3 Fred and Pamela Buffett Cancer Center, University of Nebraska Medical Center, Omaha, NE USA; 4 Eppley Institute for Research in Cancer and Allied Diseases, University of Nebraska Medical Center, Omaha, NE USA

**Keywords:** Mucins, MUC5AC, CD44, β-Catenin, Colon cancer, Colorectal cancer, 5′-fluorouracil, Chemoresistance

## Abstract

**Background:**

Differential expression of mucins has been associated with several cancers including colorectal cancer (CRC). In normal physiological conditions, secretory mucin MUC5AC is not expressed in the colonic mucosa, whereas its aberrant expression is observed during development of colon cancer and its precursor lesions. To date, the molecular mechanism of MUC5AC in CRC progression and drug resistance remains obscure.

**Methods:**

MUC5AC expression was determined in colon tissue microarray by immunohistochemistry. A RNA interference and CRISPR/Cas9-mediated system was used to knockdown/knockout the MUC5AC in CRC cell lines to delineate its role in CRC tumorigenesis using in vitro functional assays and in vivo (sub-cutaneous and colon orthotopic) mouse models. Finally, CRC cell lines and xenograft models were used to identify the mechanism of action of MUC5AC.

**Results:**

Overexpression of MUC5AC is observed in CRC patient tissues and cell lines. MUC5AC expression resulted in enhanced cell invasion and migration, and decreased apoptosis of CRC cells. MUC5AC interacted with CD44 physically, which was accompanied by the activation of Src signaling. Further, the presence of MUC5AC resulted in enhanced tumorigenesis and appearance of metastatic lesions in orthotopic mouse model. Additionally, up-regulation of MUC5AC resulted in resistance to 5-fluorouracil (5-FU) and oxaliplatin, and its knockout increased sensitivity to these drugs. Finally, we observed that up-regulation of MUC5AC conferred resistance to 5-FU through down-regulation of p53 and its target gene *p21* and up-regulation of β-catenin and its target genes *CD44* and *Lgr5.*

**Conclusion:**

Our findings suggest that differential expression of secretory mucin MUC5AC results in enhanced tumorigenesis and also confers chemoresistance via CD44/β-catenin/p53/p21 signaling.

## Background

Colorectal cancer (CRC) is the most commonly diagnosed cancer and the third leading cause of cancer-related deaths [1]. Mucins are high molecular weight glycoproteins involved in protection of the epithelial linings of different organs from physical, chemical and pathogenic insults [2]. Mucin expression is tissue specific, however, their aberrant expression is observed in multiple malignancies. Differential expression of mucins has been associated with tumor cell proliferation, migration, invasion, adhesion, and metastasis [3, 4]. In addition, alteration of mucin expression and glycosylation pattern have strongly been associated with development of CRC [3]. Interestingly, secreted mucins MUC5AC and MUC6 are not expressed in normal colonic mucosa, but both are expressed during CRC progression [2]. Development of CRC is also accompanied by loss of conventional colonic mucin MUC2 and appearance of gastric mucin MUC5AC. Loss of MUC2 expression in colon adenocarcinoma has been strongly associated with poor prognosis in stage II and III CRC patients [5]. Further studies suggest that loss of MUC2 contributes to cancer invasion and metastasis through interleukin-6 signaling [6, 7]. Under normal conditions, expression of secretory mucin MUC5AC is restricted to stomach, lungs, ear, conjunctiva, nasopharynx, and gallbladder. Although MUC5AC expression is absent in normal colon, its expression increases across benign and malignant conditions [8]. Krishn et al. (2016) observed the down-regulation of MUC2 and MUC4, and overexpression of MUC1 and MUC5AC in colonic polyps, adenomas and CRC [3]. Additionally, MUC5AC alone or in combination with CA19–9 emerged as a potential marker to discriminate pancreatic cancer from chronic pancreatitis [9]. However, to date the molecular mechanism of MUC5AC in CRC progression and drug resistance remains obscure.

Cancer cells which express certain type of mucins (MUC1, 4, 5 AC, 6, 13, 16 and 20) are resistant to chemotherapeutic drugs due to the physiological mucin barrier, drug efflux proteins, resistance to apoptosis, stem cells, and transition from epithelial to mesenchymal phenotype [10, 11]. Considering differential expression of mucins during disease development and chemoresistance, the present study explores the functional and mechanistic role of MUC5AC in CRC progression and chemoresistance. We observed physical interaction of MUC5AC with CD44 and direct impact of MUC5AC on Src signaling. Upregulation of MUC5AC expression was observed upon 5-fluorouracil (5-FU) treatment, and inhibition of MUC5AC enhanced tumor cells sensitivity to 5-FU. The resistance to 5-FU is due to down-regulation of p53 and its target molecule p21, which results in inhibition of drug-induced apoptotic cell death. Importantly, decreased expression of p53 occurs via activation of the β-catenin pathway and up-regulation of its target genes *CD44* and *Lgr5*.

## Methods

### Cell lines and culture

Human colorectal cancer cell lines (HCT-8 and LS174T) and normal colon cell line (CCD 841 CoN, ATCC® CRL1790™) were obtained from American Type Culture Collection and cultured in DMEM and MEM supplemented with 10% fetal bovine serum (FBS) and antibiotics (100 U/ml penicillin and 0.1 mg/ml streptomycin) at 37 °C with 5% CO_2_ in a humidified atmosphere. Both HCT-8 and LS174T cell lines have mutant KRAS and wild-type TP53 [12, 13]. All the cell lines were tested for mycoplasma contamination before use and validated by short tandem repeat profiling.

### Gene silencing by shRNA or siRNA transfection

For stable transfection, endogenous MUC5AC expression was silenced by a small hairpin RNA construct (pSUPER-Retro-shMUC5AC) as mentioned previously [14]. Briefly, scrambled and shMUC5AC constructs were transfected with Lipofectamine 2000 (Invitrogen, CA, USA) into phoenix cells and incubated for 48 h. Later, retrovirus titer in culture supernatant was collected and incubated in a six-well plate containing colon cell lines in the presence of hexadimethrine bromide (polybrene). After 48 h, positively infected cells were selected using puromycin at 4 μg/ml. In case of CD44 transient silencing, we used ON-TARGETplus human CD44 siRNA at 80 nM (Dharmacon, Illinois, USA) according to the manufacturer’s instructions.

### Western blot analysis

Total protein lysates were prepared in radio immunoprecipitation assay (RIPA) lysis buffer containing 50 mM Tris-HCl, 150 mM NaCl, 1% NP-40, 0.5% sodium deoxycholate, 0.1% sodium dodecyl sulfate along with protease inhibitor cocktail (Roche Diagnostics, Mannheim, Germany). Proteins (concentration from 20 to 40 μg) were resolved in 10–12% sodium dodecyl sulfate–polyacrylamide gel electrophoresis. Due to its high molecular weight, MUC5AC was resolved in 2% SDS-agarose gel. Blotting was done from gel to polyvinylidene difluoride (PVDF) membranes, and the membrane was blocked for 1 h in 5% non-fat dry milk having phosphate-buffered saline with 0.1% Tween 20 (PBST). After blocking, membranes were washed with PBST and incubated with primary antibodies at 4 °C overnight. Further, membranes were washed in PBST (3 times, 10 min each) then probed with respective secondary antibodies for 1 h at room temperature (RT) and visualized with a chemiluminescence reagent (GE Healthcare Bio-Sciences, PA, USA). The following antibodies from various vendors were used for immunoblotting studies: MUC5AC (Millipore #MAB2011, MA, USA), anti-pSrc^Y416^ (Cat. #2101), Src (Cat. #2108), anti-CD44 (Cat. #3570), cleaved PARP (Cat. #9542), cleaved caspase 9 (Cat. #20750), p21 (Cat. #2947) and β-catenin (Cat. #19807), pAKT^S473^ (Cat. #4060), AKT (Cat. #2920), Lrp6 (Cat. #2560), and CD133 (Cat. #5860) from Cell signaling Technology Inc. (MA, USA); paxillin (sc-365,174), p53 (sc-126), Chk1 (sc-8408), and β-actin (sc-47,778) from Santa Cruz Biotechnology Inc. (CA, USA); anti-integrin β4 (Cat. #ab29042) from Abcam (MA, USA); Lgr5 (Cat. #PA5–49691) from ThermoFisher Scientific (NY, USA); and MUC4 (8G7; generated in-house) [15].

### RNA isolation and real-time PCR

Total RNA was isolated from colon cell lines by using Qiagen Kit (Germantown, MD), and 2000 ng of RNA was used for the cDNA synthesis by using reverse transcriptase SuperScript®II (Invitrogen, CA, USA). SYBR Green was used for real-time PCR detection and normalized with β-actin. All samples were analyzed in triplicate and the data were calculated according to the 2^−ΔΔCT^ method.

### Immunofluorescence

For immunofluorescence, HCT-8 and LS174T cells with scrambled (Scr) vector control or MUC5AC knockdown (Sh5AC) were seeded (10,000 cells/well) in a six-well plate having sterilized cover slips and incubated for 48 h. After that, cells were washed in Hanks buffer solution (2 times, 5 min each), fixed in ice-cold methanol (− 20 °C) for 2 min and washed in PBS (2 × 5′ each). Cells were then blocked with 10% normal goat serum (Jackson Immunoresearch Labs, Inc., PA, USA) for 1 h, exposed to primary antibody (MUC5AC 1:500, Millipore sigma, USA) after PBS wash and kept at 4 °C overnight. Fluorescein isothiocyanate-conjugated anti-mouse (1:400, ThermoFisher Scientific, NY, USA) secondary antibody was added to the cells followed by 1 h incubation at RT. Finally, cells were washed with PBS (4 × 5′ each), and coverslips with adhered cells were carefully inverted on top of the microscopic slides containing anti-fade Vectashield mounting medium (Vector Laboratories, Burlingame, CA, USA) along with 4′,6-diamidino-2-phenylindole (DAPI) for Laser confocal microscopy by using an LSM 510 microscope (Carl Zeiss GmbH, Germany).

### Cell proliferation and colony formation assay

Sh5AC or Scr cells were seeded (5000 cells/well) in 96-well plates containing DMEM, 1% FBS with puromycin selection, and MTT assay was performed for six days. Each day, MTT reagent was added and incubated for 4 h followed by dissolving formazan crystals in DMSO, and absorbance was recorded. For colony formation assay, approximately 2500 cells were seeded in a six-well plate, after 15–18 days cells were stained with crystal violet, further dissolved in 10% (v/v) acetic acid, and absorbance was recorded at 590 nm [16].

### Cell migration and invasion assays

Both Scr or Sh5AC cells (1 × 10^6^) were seeded on top of a Boyden Chamber or matrigel insert (8 μm, Corning® BioCoat™ Matrigel® Invasion Chamber) containing serum-free medium for cell migration or invasion assays, respectively; 20% FBS in DMEM was added in the lower chamber of the 6-well plate, and cells were incubated for 24 h. The invaded cells in the lower side of the membrane were stained with a Diff-Quick stain, and the number of migrated or invaded cells was counted in different fields.

### CRISPR/Cas9-driven *MUC5AC* gene knockout in colon cell lines

The genomic deletion of MUC5AC in colon cell lines (HCT-8 and LS174T) was carried out by using CRISPR/Cas9 vector, pD1401-AD (ATUM, CA, USA), with a specific MUC5AC guide RNA sequence (1st gRNA: 5′-CCGAATCCAGCTACAAGCAC-3′, 2nd gRNA: 5′-TGCCCTCTCTCCTATCGCCC-3′). After 48 h of transfection, single cells were sorted by FACS based on GFP expression into a 96-well plate to obtain MUC5AC knockout (KO) clones. The complete KO was confirmed by western blotting using anti-MUC5AC antibody.

### Isolation of side or stem cell population

Stem cell or non-stem cell populations were isolated by using ATP-binding cassette inhibitor (verapamil) and DNA staining dye (Hoechst 33342) [17]. Both parental and MUC5AC KO clones were seeded (approximately 1 × 10^6^ cells) and treated with verapamil (75 μM). After incubation at 37 °C for 15 min, Hoechst 33342 dye was added (5 μg/ml) and incubated for 90 min in the dark, and FACS sorting was carried out. After sorting, stem cell population (SP) cells were seeded in 0.1% gelatin-coated plates containing DMEM-F12 medium along with stem cell growth factors [18], whereas non-stem cell population (NSP) cells were grown in regular DMEM medium as describe above.

### Tumor spheroid assay

Isolated SP from HCT-8 parental or MUC5AC KO clones were seeded (5000 cells/well) in DMEM-F12 with B27 supplement spheroid media [18] in a 96-well low-attachment plate. After 3 days, tumor spheroids of both parental and KO clones were analyzed.

### Immunoprecipitation

Interaction of MUC5AC and CD44 was assessed by a co-immunoprecipitation assay. Protein A/G plus agarose beads (Santa Cruz Biotechnology, TX, USA) were pre-incubated with a protein sample (1 mg/ml) for 1 h followed by washing and centrifugation. The next day, beads were incubated with MUC5AC antibody (3 μg) and IgG isotype control (3 μg) along with pre-cleared protein samples at 4 °C in a rotary shaker for 5–6 h. After that, samples were centrifuged at 3000 rpm for 3 min to remove supernatant, the pellet was washed with RIPA buffer (4 × 3 min), and pulldown was assessed using 2% SDS-agarose gel electrophoresis along with input (3–4% of the total protein) and IgG control samples in Laemmli buffer. For co-immunoprecipitation, protein samples were resolved in 10% SDS-PAGE, transferred onto a PVDF membrane, probed with primary anti-CD44 antibody, and incubated at 4 °C overnight after blocking with 5% non-fat dry milk in PBST. Next, the membrane was washed with PBST (3 × 10 min), probed with the secondary antibody for 1 h at RT, and visualized with chemiluminescence ECL reagent. Co-localization of MUC5AC and CD44 was done by immunofluorescence in colon cells (HCT-8 and LS174T) as mentioned in the previous section by using anti-mouse MUC5AC and anti-rabbit CD44 (KO601, Trans Genic Inc. Kobe, Japan) antibodies.

### Preparation of MUC5AC-conditioned media

Both parental and MUC5AC KO cell lines were cultured in DMEM supplemented with 2% FBS for 48 h. After 48 h, conditioned media (CM) was collected and centrifuged at 300 x*g* for 2 min to remove cell debris. The supernatant of centrifuged CM fraction was filtered through a 0.2 μm Nylon sterile syringe filter and concentrated using Amicon® Ultra 15 mL Centrifugal Filters (100 kDa NMWL), and MUC5AC KO cells were incubated with CM.

### Sandwich ELISA for MUC5AC

The measurement of MUC5AC concentration before or after CM preparation was done by ELISA. Briefly, MUC5AC (1-13 M1) capture antibody (2 μg/ml in carbonate buffer) was coated in 96-well plates (100 μl/well) and incubated at RT overnight. The next day, coated plate was washed twice with PBST, and blocking was done with 3% BSA for 3 h at 37 °C. After blocking, the plate was washed three times with PBST, and samples (1:5 dilution) or standards (100 μl/well) were added in triplicate followed by overnight incubation at 4 °C. Then biotinylated detection antibody (45 M1, Thermo Fisher Scientific, USA) was added and incubated for 2 h at RT after washing again with PBST. Next, polymeric streptavidin-HRP was added and incubated for 30 min at RT. After incubation, freshly prepared 3,3′,5,5′-tetramethyl benzidine (Sigma–Aldrich, Inc., USA) substrate solution was added and incubated for 20 min in the dark. The enzymatic reaction was stopped by adding 1 M sulfuric acid, and the plate was read at 540 nm.

### Cell cycle analysis

Both parental and KO clones of HCT-8 cell lines (1 × 10^6^ cells) were seeded in serum free media and treated with IC_50_ of 5-FU (5 μM) for 48 h. After treatments, cells were fixed overnight at 4 °C in 70% ethanol. Later, cells were washed with PBS and stained with Telford reagent (50 μg/mL propidium iodide, 90 mM EDTA, 0.1% Triton X-100, and 1 μg/mL RNase A) for 1 h at 4 °C. The DNA content of stained cells were analyzed by using a FACS flow cytometer.

### In vivo tumorigenesis

For tumorigenesis, Scr or Sh5AC (1 × 10^6^) cells in PBS were injected subcutaneously into athymic nude mice (age 5–6 weeks), and every two days tumor volume was measured with a digital caliper. At the end of the study, mice were sacrificed and tumors were resected and weighed. Further, proteins were isolated from both Scr- and Sh5AC-pooled tumors and used for western blot analysis. In the case of the orthotopic mouse model, parental and MUC5AC knockout (CRISPR/Cas9) HCT-8 cell lines were infected with firefly luciferase + IRES-eGFP Lentifect™ purified lentiviral particles (GeneCopoeia, MD, USA). After 48 h transduction, GFP-positive cells were FACS sorted and resuspended in DMEM. Before injecting, luciferase-positive cells were visualized under an IVIS imaging system using D-luciferin (Caliper life sciences, Hopkinton, MA) substrate. For in vivo experiment, 2 × 10^6^ viable cells in PBS were injected into the caecal submucosa of the athymic nude mice after laparotomy in the presence of ketamine/xylazine (87.5 mg/12.5 mg/kg) anesthesia. Tumor growth was monitored by bioluminescence imaging using the IVIS system after injecting D-luciferin (150 mg/kg) intraperitoneally. After imaging several time points, animals were euthanized due to heavy tumor burden in the MUC5AC-expressing group. All the animals were maintained in accordance with guidelines and protocols approved by the Institutional Animal Care and Use Committees (IACUC) of the University of Nebraska Medical Center, Omaha, Nebraska and USA.

### Immunohistochemistry

A tissue microarray (TMA-CO2081) of human colon cancer was purchased from US Biomax Inc. (MD, USA). Tissue slides were baked in the oven at 58 °C overnight. The next day, slides were cooled to RT and washed using xylene (4 × 10 min) to remove paraffin and hydrated through graded alcohol solution (100, 90, 70, 50, 30, and 20%) for 10 min each. Further, quenching of endogenous peroxidase activity (3% H_2_O_2_ in methanol) was carried out by incubation for 1 h in the dark. It was followed by heat-induced antigen retrieval in 0.01 M citrate buffer in the microwave for 15 min. Next, blocking was done by using 2.5% horse serum (Impress Reagent Kit, Vector Laboratories, CA, USA) after washing the slides to remove excess citrate buffer. The tissue sections were further incubated with primary antibodies for MUC5AC (45 M1, 1:100 dilutions) and CD44 (1:60 dilutions) at 4 °C overnight. After washing with PBST (4 × 10 min), slides were probed with HRP-labelled universal anti-mouse/rabbit IgG for 30 min and then stained with DAB substrate kit (Vector Laboratories, Burlingame, CA, USA). Then slides were counterstained with hematoxylin, dehydrated with different graded alcohols followed by xylene washes, and mounted with Permount mounting medium (Fisher Scientific, Grand Island, NY, USA) capped by a coverslip. Composite scoring for MUC5AC was carried by a pathologist and is the product of intensity score and percentage positivity of cells for the given marker. Intensity score for MUC5AC was done on a scale of 0–3 (0-negative, 1-weak, 2-moderate, 3-intense staining) and the percent of positive cells was given on a scale of 1–4 (1: 0–25%; 2: 26–50%; 3: 51–75%; and 4: 76–100%) [3].

### Statistical analysis

All the data are presented as means with their standard error of the mean (means + SEM). Analyses were performed by using GraphPad Prism Software 7.0 (San Diego, CA, USA). Student t-test were used for two groups, and One-way ANOVA was applied for comparing more than two groups by Tukey’s post-hoc test. ^*^p < 0.05, ^**^p < 0.01 and ^***^p < 0.001 were considered statistically significant.

## Results

### MUC5AC expression is up-regulated in human colon cancer tissues and cell lines

**Fig. 1:**
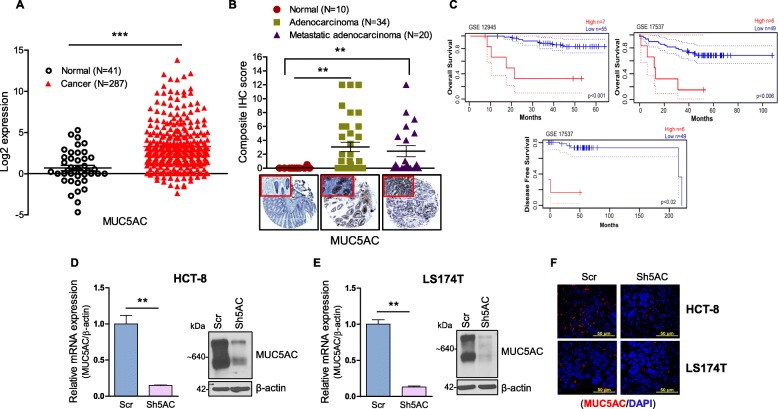
Elevated expression of MUC5AC in colorectal cancer patients and cell lines. **a** Expression of MUC5AC in colorectal cancer (CRC) patients (*N* = 287) and normal (*N* = 41) cases was analyzed in The Cancer Genome Atlas Program (TCGA) colorectal adenocarcinoma (COAD) dataset. A statistically significant difference was observed between normal vs cancer (^***^p < 0.001). **b** Immunohistochemistry staining of MUC5AC in a commercially available CRC tissue microarray (US Biomax, Rockville, United States). MUC5AC-positive staining is represented as a composite score. Representative images of differential MUC5AC staining in normal (*N* = 10), adenocarcinoma (*N* = 34, ^**^p < 0.01), and metastatic adenocarcinoma (*N* = 20, ^**^p < 0.01). **c** High expression of MUC5AC is associated with a decrease in overall and disease-free survival in CRC patients. **d** and **e** Quantitative real-time PCR and immunoblotting images showing decreased levels of MUC5AC expression in HCT-8 and LS174T cells. **f** Confocal images showing reduction in the MUC5AC expression in Sh5AC cell lines of both HCT-8 and LS174T by using MUC5AC primary antibody and stained with Alex Fluor secondary antibody (Red). Nuclear staining was carried out with DAPI (blue)

Our aim was to study whether MUC5AC had a role in colon cancer progression. We initially analyzed The Cancer Genome Atlas (TCGA-COAD) database containing 287 colon cancer and 41 normal samples for MUC5AC expression and observed a significant (*p* < 0.05) up-regulation of MUC5AC in colon cancer patients (Fig. 1a). Next, we examined MUC5AC expression in colon TMA, which showed a higher expression of secretory mucin MUC5AC protein in both adenocarcinoma (*n* = 34, *p* < 0.01) and metastatic adenocarcinoma (*n* = 20, p < 0.01) patient tissues (Fig. 1b). To assess the significance of MUC5AC expression in CRC patient survival, publicly available datasets (GEO accession: GSE12945 and GSE17537) were analyzed for MUC5AC expression (http://dna00.bio.kyutech.ac.jp/PrognoScan/index.html). Kaplan-Meier analysis showed a significant (*p* < 0.001 and *p* < 0.006) decrease in overall as well as disease-free survival in patients with high MUC5AC expression, suggesting its tumor-promoting role in CRC (Fig. 1c).

### MUC5AC enhances proliferation, invasion, and migration of CRC cells in vitro

Initially, we screened for MUC5AC expression in normal (CCD 841 CoN) as well as CRC cell lines (HCT-8 and LS174T). Interestingly, we observed higher MUC5AC expression in CRC cell lines as compared to normal cell line (Figure S1A). Next, to study the functional role of MUC5AC in colon cancer progression, endogenous expression of MUC5AC was knocked down in the above mentioned CRC cell lines. The stable knockdown of MUC5AC (Sh5AC) was performed by using the small hairpin RNA system. As shown in Fig. 1d and e, knockdown of MUC5AC in both HCT-8 and LS174T cell lines was confirmed at mRNA and protein levels (~ 80–90%) by qRT-PCR and immunoblot, respectively. Further, we also observed MUC5AC expression by confocal microscopy and confirmed that its expression was reduced in Sh5AC cells as compared to Scr (Fig. 1f). In addition, Sh5AC cells showed less cell proliferation (Figure S1B and C), invasion, and migration (Figure S1D and E) compared with Scr cells, which suggests that MUC5AC impacts in vitro colon cancer cell growth, migration, and invasion properties.

### Isolated cancer stem cells or side population shows higher MUC5AC and CD44 expression

**Fig. 2:**
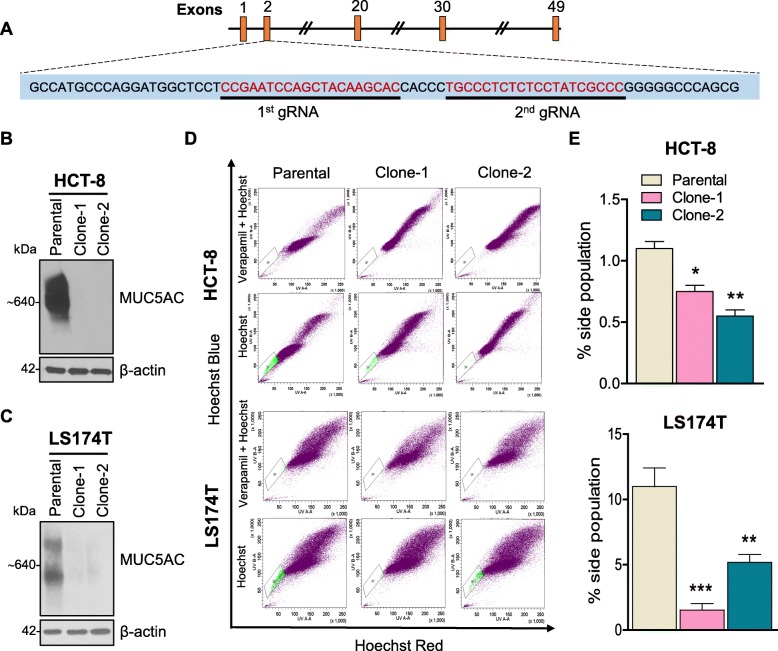
MUC5AC-expressing colon cancer cells exhibit a higher percentage of stem cells or side population**. a** Schematic depicting the location of gRNAs (at exon 2) of CRISPR/Cas9 used for knocking out the *MUC5AC* gene. **b** and **c** Immunoblot showing deletion of MUC5AC in clones of CRC cell lines (HCT-8 and LS174T). **d** and **e** Side or stem cell population (SP) was isolated in the presence of an ATP-binding cassette inhibitor (verapamil). A higher SP percentage was observed in HCT-8 and LS174T parental cells as compared to MUC5AC knockout tumor cells (^*^p < 0.05 and ^**^p < 0.01)

**Fig. 3:**
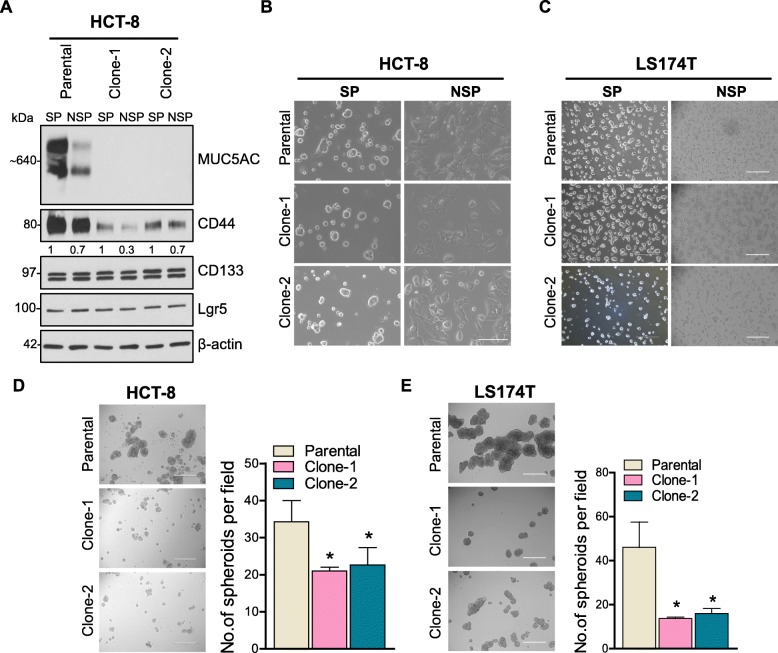
MUC5AC expression results in the maintenance of a stem-like phenotype in colon cancer cells. **a** Side or stem cell population (SP) cells were isolated based on Hoechst exclusion staining via FACS and expanded by culturing the cells in the specific stem cell growth media. Western blot analysis showed an increase in MUC5AC and CD44 levels in parental SP cells. **b** and **c** Morphology analyses of SP and non-side population (NSP) of parental and KO clones isolated from HCT-8 and LS174T cells. **d** and **e** Formation of colon spheres/tumor spheres in parental and KO clones (Clone-1 and -2) from HCT-8 and LS174T after 10 days. A significant decrease in spheroid formation was observed in KO clones as compared to parental cells (*p < 0.05)

To understand the molecular mechanism of MUC5AC in CRC progression, we used the CRISPR/Cas9 genetic modification system to delete the *MUC5AC* gene by designing a specific guide RNAs at exon 2 (Fig. 2a). As shown in Fig. 2b and c, complete absence of the MUC5AC protein was observed in two KO clones by western blotting as compared to parental HCT-8 and LS174T cell lines. Recent studies directly implicate a subset of the population within the tumor, known as stem cells or side population in mediating tumor recurrence and also resistance to chemotherapeutic drugs [19]. Therefore, we isolated SP cells from parental as well as MUC5AC KO clones and observed that the percentage of SP was higher in parental cells (1.1% in HCT-8 and 7.5% in LS174T) as compared to KO clones (0.7 and 0.5% in clones 1 and 2 of HCT-8, and 0.3 and 2.4% in clones 1 and 2 of LS174T) (Fig. 2d and e). Interestingly, we also observed elevated MUC5AC expression in isolated SP as compared to NSP of the parental cell line (Fig. 3a). Similarly, both parental and KO-SP clones showed higher CD44 expression compared to NSP (Fig. 3a). However, we did not observe down-regulation of other stem cell markers (CD133 and Lgr5) (Fig. 3a), suggesting that both MUC5AC and CD44 have a positive role in maintaining a specific subset of the cancer stem cell population.

The presence of MUC5AC in isolated SP from parental and KO clones of both HCT-8 and LS174T showed distinct morphology as compared to NSP cell lines (Fig. 3b and c). In addition, we observed a significant increase (*p* < 0.05) in the number of spheres formed in SP of both parental cells as compared to KO clones (Fig. 3d and e), thereby suggesting that MUC5AC helps in maintaining the stemness and colonosphere-forming ability of SP cells.

### Secretory MUC5AC mediates CD44 expression

**Fig. 4:**
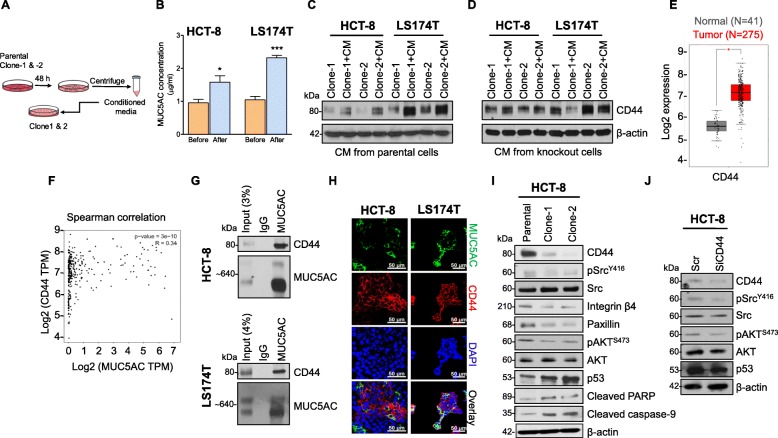
MUC5AC physically interacts with CD44 to accelerate cellular signaling via Src. **a** Schematic representation of conditioned media (CM) collected from parental and KO clones (Clone-1 and -2) of HCT-8 and LS174T cell lines. **b** MUC5AC concentration is measured before or after CM preparation by ELISA. **c** and **d** Immunoblotting of CD44 from KO clones (Clone-1 and -2) treated with CM collected from parental cell lines (HCT-8 and LS174T) showed higher expression of CD44 in the presence of CM compared with untreated cells. No difference in CD44 expression was observed in KO clones supplemented with conditioned medium from KO clones. **e** Significantly higher expression of CD44 was observed in tumor patients (*N* = 275) compared with healthy controls (N = 41) in TCGA-COAD (^*^p < 0.05). **f** MUC5AC and CD44 correlated positively in the TCGA-COAD dataset. **g** Immunoblot showed a physical interaction of MUC5AC-CD44 in CRC cell lines. **h** Both HCT-8 and LS174T cell lines co-stained with MUC5AC and CD44 antibodies. FITC-labelled and Alexa Fluor-labelled secondary antibodies were used for MUC5AC (Green) and CD44 (Red) staining, respectively. DAPI was used for nuclei staining. **i** Interaction of MUC5AC with CD44 mediates activation of various down-stream signaling molecules assessed in whole cell lysates isolated from parental and KO clones (Clone-1 and -2) of HCT-8. **j** Transient knock-down of CD44 altered its down-stream signaling molecules

Conditioned media (CM) was collected from both parental as well as KO clones and only KO clones were incubated  with this CM for 48 h (Fig. 4a). As shown in Fig. 4b, before treatment, MUC5AC concentration was significantly enriched in both parental cell lines, as measured by ELISA. After treatment with CM from parental cells, both KO clones expressed higher CD44 as compared with untreated cells (Fig. 4c). Secondly, to rule out the other factors in CM facilitating CD44 expression, we incubated KO clones with CM derived from MUC5AC KO cells for 48 h. No difference in CD44 expression in the presence or absence of CM (Fig. 4d) was observed, suggesting that secretory MUC5AC has a potential role in modulating CD44 expression.

### Physical interactions of MUC5AC with CD44 drives invasion and migration

The above results suggest a strong correlation between MUC5AC and CD44 expression. Hence, we analyzed the TCGA database containing 275 colon cancer and 41 normal samples for correlation between CD44 and MUC5AC expression (http://gepia.cancer-pku.cn/). Significant up-regulation of CD44 (Fig. 4e) and a strong correlation of MUC5AC with CD44 was observed in CRC patient tissue datasets (Fig. 4f). Next, we observed a strong physical interaction and co-localization between MUC5AC and CD44 by immunoprecipitation and confocal microscopy, respectively in both CRC cell lines (Fig. 4g and h). CD44 is a transmembrane glycoprotein involved in several cellular processes like growth, invasion, and migration through Src signaling [20, 21]. In our study, we observed that knockdown of MUC5AC decreased the expression of cell migration/invasion and survival molecules such as phosphorylated Src, paxillin, integrin-β4, and phosphorylated AKT, and increased pro-apoptotic molecules like cleaved PARP and cleaved caspase-9 along with tumor suppressor p53 (Fig. 4i). Transient knockdown of CD44 (siRNA 80 nM) in the HCT-8 cell line decreased the expression of down-stream signaling molecules, such as Src and AKT, suggesting that the MUC5AC interaction with CD44 mediated via Src signaling (Fig. 4j).

### MUC5AC promotes tumorigenesis through the CD44-Src-integrin axis

**Fig. 5:**
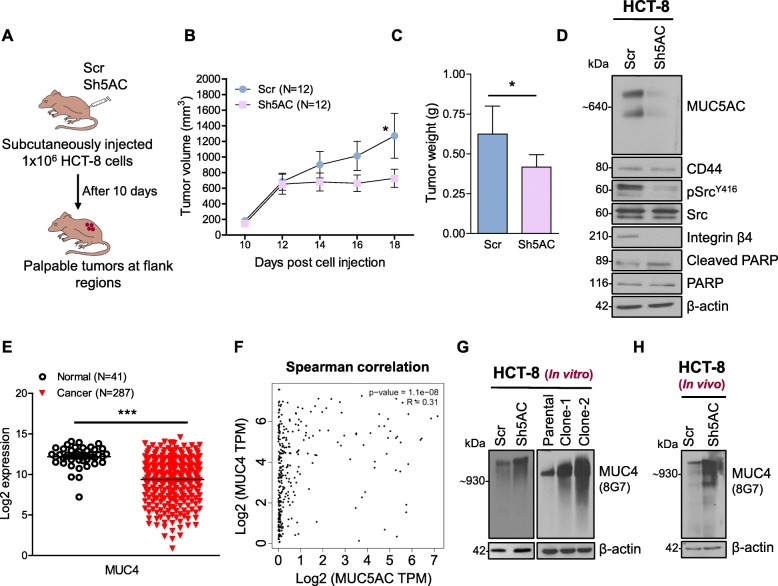
Decreased tumor burden in mouse model injected subcutaneously with MUC5AC knockdown cell line. **a** Schematic representation of subcutaneous injection of HCT-8 scrambled (Scr) and MUC5AC knockdown (Sh5AC) cells in athymic nude mice. **b** and **c** Mice injected with Sh5AC cell lines exhibited decreased tumor volume and weight as compared to Scr animals. **d** Immunoblotting of various signaling molecules associated with MUC5AC-CD44 interaction in subcutaneous tumor tissue lysate. **e** Expression of transmembrane mucin MUC4 was lower in colon cancer patients compared with healthy controls. **f** Correlation between MUC4 and MUC5AC mRNA expression in colon cancer patients (TCGA-COAD dataset). **g** and **h** In vitro knockdown and knockout of MUC5AC showed an increase in MUC4 expression (analyzed by anti-MUC4 8G7 antibody). Tumor tissues from mice subcutaneously injected with Sh5AC cells also expressed higher MUC4 as compared to Scr group

Subcutaneous implantation of both Scr and Sh5AC cell lines in the flank region of athymic nude mice induced palpable tumors after 10 days (Fig. 5a). The mice with Scr-MUC5AC cell lines showed a significant increase in tumor volume (Fig. 5b) as well as tumor weight compared to the Sh5AC group (Fig. 5c). Based on western blot analysis, we observed that Sh5AC animals had a lower expression of MUC5AC as compared to the Scr (Fig. 5d). Down-regulation of MUC5AC also reduced CD44 expression and its cell migratory down-stream target Src along with integrin-β4 (Fig. 5d). The reduction in CD44 levels in Sh5AC tumors was not as significant as in cell lines, possibly due to the mixed origin of lysate from both tumor and normal epithelial cells. In addition, less tumor growth was observed in the Sh5AC group, which might be partially due to up-regulation of cleaved PARP as an apoptotic marker (Fig. 5d).

### Loss of MUC5AC promotes MUC4 expression in CRC

In our previous study, we demonstrated that MUC4 expression is reduced in CRC cell lines by Notch effector *Hath1* through the Wnt/β-catenin pathway [22]. Here, we showed that the expression of MUC4 in the TCGA-COAD database is significantly down-regulated (Fig. 5e) and a spearman correlation showed a correlation between MUC4 and MUC5AC (http://gepia.cancer-pku.cn/) in CRC patients (Fig. 5f). In the current study, both knockdown and KO of MUC5AC in the HCT-8 cell line showed up-regulation of MUC4 expression (Fig. 5g), and similar results were observed in the subcutaneously injected Sh5AC animal group (Fig. 5h). Nevertheless, the role of MUC4 expression in CRC is still controversial and further studies are needed in this area.

### Orthotopic implantation of MUC5AC promotes tumor growth and metastasis

**Fig. 6:**
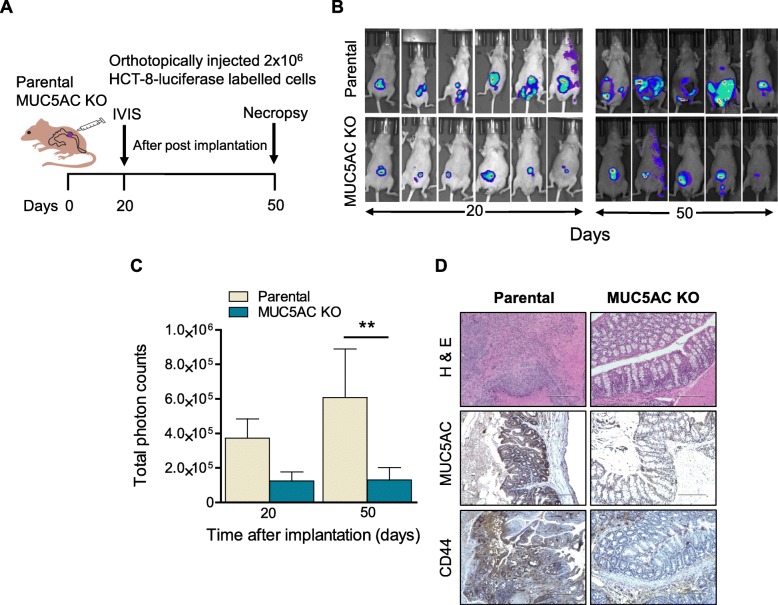
MUC5AC expression increases tumor burden in orthotopic mouse model. **a** Schematic representation of colon orthotopic mouse model. Approximately 2 × 10^6^ cells (HCT-8 parental and MUC5AC knockout, labelled with luciferase) were injected in the serosa of the caecal region. **b** and **c** In vivo tumor growth was imaged using the IVIS imaging system after intraperitoneal injection of D-luciferin (150 mg/kg) at days 20 and 50. Photon counts from both parental and KO clones were quantified 20 and 50 days post-implantation. **d** Representative hematoxylin and eosin stain of parental and KO cells injected orthotopically in athymic nude mice. Higher tumor burden was observed in the colon of mice injected with parental cells

To study the role of MUC5AC in CRC progression and metastasis, we injected a luciferase-labelled HCT-8 parental as well as MUC5AC KO cell lines in the caecal region of athymic nude mice (Fig. 6a). After 20 days of laparotomy, tumor growth was monitored by an IVIS imaging system after injecting D-luciferin substrate intraperitoneally. Higher tumor burden was observed in the MUC5AC-expressing parental group at both 20 and 50 days as compared to animals bearing KO cells (Fig. 6b and c). During the experimental period, one animal from each group was sacrificed due to heavy tumor burden. Further, the presence of MUC5AC led to more invasive tumors with higher expression of MUC5AC and CD44 in colon tissue (Fig. 6d). Finally, in the presence of MUC5AC, metastatic lesions were observed in the peritoneum, liver, spleen, stomach, and kidney; however, in the KO group, only peritoneal metastasis was observed (Figure S2).

### Presence of MUC5AC mediates resistance to 5-FU

**Fig. 7:**
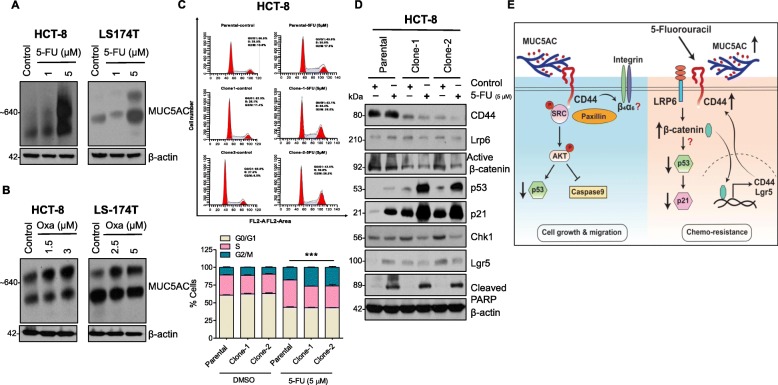
Presence of MUC5AC mediates resistance to 5-FU. **a** 5-FU treatment at 1 and 5 μM for 48 h increased expression of MUC5AC in HCT-8 and LS174T cell lines. **b** Similarly, in the presence of oxaliplatin both cell lines showed upregulation of MUC5AC expression. **c** DNA content in different phases of the cell cycle was assessed by propidium iodide staining of parental and MUC5AC knockout clones (Clone-1 and -2) followed by FACS analysis. Cell cycle analysis showed that in the absence of MUC5AC, cells were arrested at G2/M phase. **d** The presence of MUC5AC facilitates 5-FU resistance via the β-catenin/p53/p21 axis and via upregulation of β-catenin target genes *Lgr5* and *CD44*. **e** Schematic diagram showing MUC5AC mediates CRC cell survival, migration, and invasion and confers 5-FU resistance via Src and β-catenin signaling, respectively

CRC often recurs in patients because of its drug resistance. Several studies are suggesting that mucins impart resistance to chemotherapeutic drugs [23–25] by different mechanisms of action, such as physical barrier formation, resistance to apoptosis, drug exclusion, enrichment of stem cells, and epithelial-mesenchymal transition [19]. To determine the role of secretory mucin MUC5AC in CRC chemoresistance, we used 5-FU, which is mostly used for CRC therapy along with oxaliplatin (an alkylating agent widely used for treating advanced CRC in combination with 5-FU). 5-FU and oxaliplatin treatment increased MUC5AC expression in a dose-dependent manner in HCT-8 and LS174T cell lines (Fig. 7a and b). In addition, parental cells expressing MUC5AC showed greater resistance to 5-FU (Figure S3A) and oxaliplatin (Figure S3B), as revealed by significantly higher cell viability in parental cells than KO clones.

### MUC5AC mediates colon cancer chemoresistance through the β-catenin/p53/p21 axis

To evaluate the mechanism by which MUC5AC mediates chemoresistance in CRC, we treated the HCT-8 cell line with 5-FU (5 μM) and observed that KO clones had higher cell cycle arrest at the G2/M phase (Fig. 7c). To maintain DNA integrity in the presence of a stress condition, p53 activates its down-stream target p21 (WAF1/CIP1) to induce cell cycle arrest [26]. In the present study, treatment with 5-FU in KO clones induced higher expression of p53 and its target p21 (Fig. 7d). Additionally, due to DNA damage, Chk1 plays an important role in check point activation and mediates p53 activation via the Wnt signaling pathway [27]. Here, we observed that 5-FU treatment reduced expression of Chk1 in KO clones and arrested cells at the G2/M phase, which resulted in activation of p53 to induce apoptosis (Fig. 7d). Moreover, 5-FU treatment also up-regulated Wnt ligand co-receptor Lrp6, which induced expression of β-catenin and its target genes *CD44* and *Lgr5* (Fig. 7d), suggesting that the presence of MUC5AC facilitates 5-FU resistance via the β-catenin/p53/p21 axis.

## Discussion

Mucins are implicated in cancer cell behavior and cellular signaling pathways that result in epithelial tumor progression and resistance to chemotherapeutic drugs [19]. Studies have suggested that secretory mucin MUC5AC is overexpressed in several cancers [9, 14, 28, 29] and its up-regulation favors progression-free as well as cancer-specific survival of intermediate stages II and III of CRC patients [30]. In addition, MUC5AC enhances pancreatic cancer cell adhesion and invasion potential via up-regulation of integrins, matrix metalloproteinases, and ERK signaling [31]. However, the molecular mechanism by which MUC5AC mediates CRC progression and resistance to chemotherapy is not known. We observed that MUC5AC was overexpressed in CRC patients and cancer cell lines, and its knockdown in CRC cell lines reduced proliferation, invasion, and migratory potential, suggesting that MUC5AC has a tumor promoting role in CRC.

Multiple studies have indicated that mucins mediate chemoresistance through the cancer stem cells (CSCs) population within the tumor. In addition, tumor relapse after therapy might be due to the presence of CSCs because of their self-renewing capacity [19]. Alam et al. (2013) observed that the c-terminal domain of MUC1 up-regulates the breast CSC marker aldehyde dehydrogenase 1A1 (ALDH1A1) through ERK1 and c/EBP on the ALDH1A1 promoter [32]. Moreover, overexpression of MUC4 leads to up-regulation of the CD133 stem cell marker either directly or indirectly via HER2 in ovarian cancer [33]. We also previously observed that down-regulation of MUC4 reversed gemcitabine resistance in pancreatic cancer stem/progenitor cells [34]. However, no reports are available to date in the context of MUC5AC’s role in the CSCs of CRC. Therefore, to understand the molecular mechanism of MUC5AC in CRC progression, we deleted the *MUC5AC* gene in CRC cell lines and observed a lower percentage of SP compared with MUC5AC-expressing cells. In addition, the isolated stem cell population overexpressed MUC5AC and CD44. Moreover, our CM experiment also suggested that the presence of MUC5AC in the CRC cell secretome regulates CD44 expression, which provides a strong relationship between MUC5AC and CD44.

Previously, it was reported that CD44, a transmembrane glycoprotein, is aberrantly expressed in several cancers and regulates cancer cell migration, invasion, and metastasis by interacting with extracellular ligands [35]. It is also considered an important stem cell marker and is responsible for resistance of tumor cells to drugs [20, 36–38]. Transmembrane CD44 is a non-tyrosine receptor and transmits its signals through the oncogenic protein Src and activation of down-stream effector molecules. Recent study have shown CD44 to regulate breast cancer cell proliferation, migration, and invasion via Src signaling [21]. Alteration of mucin expression or glycosylation pattern in cancer cells modulates the interactions with other cell surface receptors. The secretory gel-forming mucin MUC5AC may interact with cell surface molecules to mediate cancer progression [39]. We observed that in the TCGA database, CRC patients had an overexpression of CD44 as compared to normal patients, and a positive correlation was observed between MUC5AC and CD44. Furthermore, KO clones expressed less CD44, suggesting a possible association between these molecules. An immunoprecipitation assay demonstrated a physical interaction of MUC5AC with CD44, along with co-localization in CRC cell lines. Additionally, we observed that the interaction of MUC5AC with CD44 impacted CRC migration and invasion via Src, integrin-β4, and paxillin signaling. Similarly, our in vivo study also suggested that lower expression of MUC5AC in subcutaneous tumors was associated with down-regulation of CD44. Overall, the study indicated that interaction of MUC5AC-CD44 might be involved in CRC cell migration and invasion.

We previously reported that the transmembrane mucin MUC4 is aberrantly expressed in different cancers [40–42], though its expression in CRC was lost in the adenoma-carcinoma sequence [3]. Additionally, reduced MUC4 expression in CRC was observed by inhibiting Notch effector Hath1 [22]. In the present study, we found that suppressing the expression of MUC5AC in cell lines as well as subcutaneous tumors led to up-regulation of MUC4, which suggested a compensatory role of these two mucins. Interestingly, we also observed that a LS174T cell line expressing very low or no MUC4 had high levels of MUC5AC in it. These studies and others suggest that mucins are useful to distinguish cancer from benign conditions [3]. Similarly, our orthotopic mouse model showed that the presence of MUC5AC enhanced tumor growth and metastasis, as corroborated in previous findings [9, 14, 28, 29].

Currently, targeting mucins in cancer cells is challenging due to resistance to chemotherapeutic drugs [11, 19]. In the present investigation, CRC cells treated with 5-FU and oxaliplatin expressed higher levels of MUC5AC. In addition, an upregulation of CD44 was also observed in HCT-8 cells upon 5-FU treatment alone. However, the links between MUC5AC and CD44 signaling in response to 5-FU treatment is not known. Previous studies have reported chemotherapeutic drugs targeting the Wnt pathway are gained clinical relevance, however, Wnt/β-catenin mediated resistance to drug remains unclear [27, 43]. Our present study showed that treatment with 5-FU increased the MUC5AC and CD44 levels that led to upregulation of Wnt ligand co-receptor, Lrp6. Accumulation of β-catenin (key component of Wnt pathway) in the nucleus results in the activation of several cell proliferating genes. During cancer, abnormal β-catenin expression is associated with inactivation of tumor suppressor *TP53* gene [44, 45]. Here, we reported that 5-FU dependent down-regulation of β-catenin resulted an increase in p53 expression and its target gene *p21* to cause cell cycle arrest and apoptosis in the absence of MUC5AC.

## Conclusion

Overall, our findings suggest that secretory mucin MUC5AC has a tumor promoting role via the transmembrane protein CD44 and confers chemoresistance via the β-catenin/p53/p21 signaling pathway in CRC (Fig. 7e).

## Supplementary information

**Additional file 1 : Figure S1.** MUC5AC mediates colon cancer cell motility, migration, and invasion. (A) MUC5AC expression was up-regulated in human CRC cell lines (HCT-8 and LS174T) as compared to a normal colon cell line (CCD 841 CoN). A549 (lung cancer cell line) was used as a positive control. (B) Cell viability of HCT-8 and LS174T cell lines stably transfected with Scr and Sh5AC was assessed by MTT assay. (C) Colony formation assay showed fewer colonies in Sh5AC cell lines compared with Scr. (D) The presence of MUC5AC in Scr cell lines increased colon cancer migration (by Boyden chamber) and invasion (by matrigel coating). (E) Sh5AC cells exhibited less cell migration than Scr cells in the wound healing assay. **Figure S2.** Animals with orthotopic implantation of parental MUC5AC cell lines showed different metastatic lesions as compared to knockout group. **Figure S3.** MUC5AC knockout sensitizes colon cancer cells to 5-FU treatment. (A and B) Cell viability was measured by MTT assay. Bar diagram indicating 5-FU and oxaliplatin treatment for 48 h significantly decreased cell viability in KO clones (Clone-1 and -2) compared with parental HCT-8 and LS174T CRC cell lines.

## Data Availability

All data generated or analyzed during this study are included in this manuscript.

## Abbreviations

5-FU: 5-fluorouracil
CM: Conditioned media
CRC: Colorectal cancer
KO: Knockout
NSP: Non-side population
SP: Side population
