# Supplementary figures and images for: Molecular implications of MUC5AC-CD44 axis in colorectal cancer progression and chemoresistance
Source: Mol Cancer. 2020 Feb 25;19:37. doi: 10.1186/s12943-020-01156-y (PMC7041280; doi:10.1186/s12943-020-01156-y)

Supplementary-1

**A**

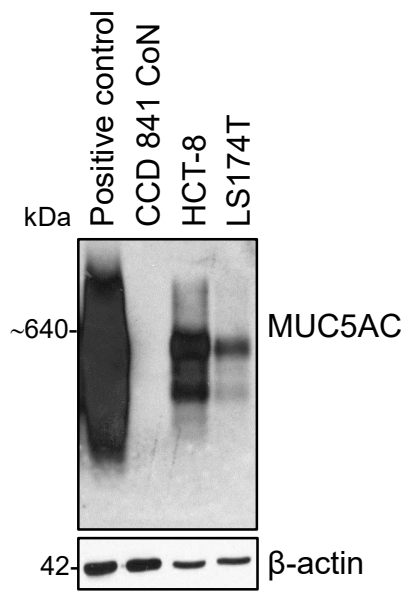

**B**

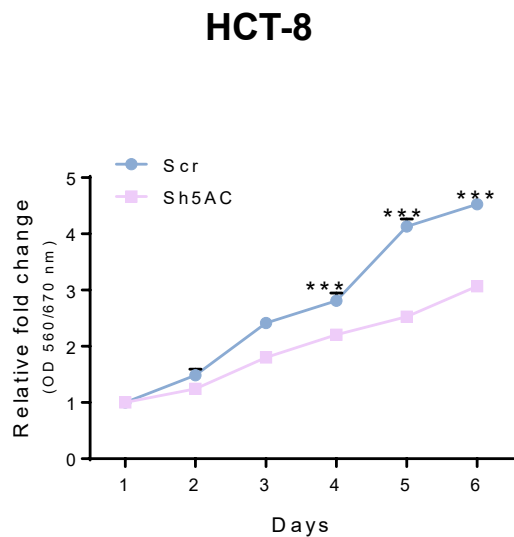

**LS174T**

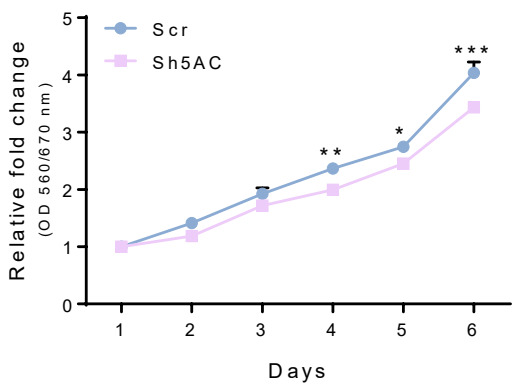

**D**

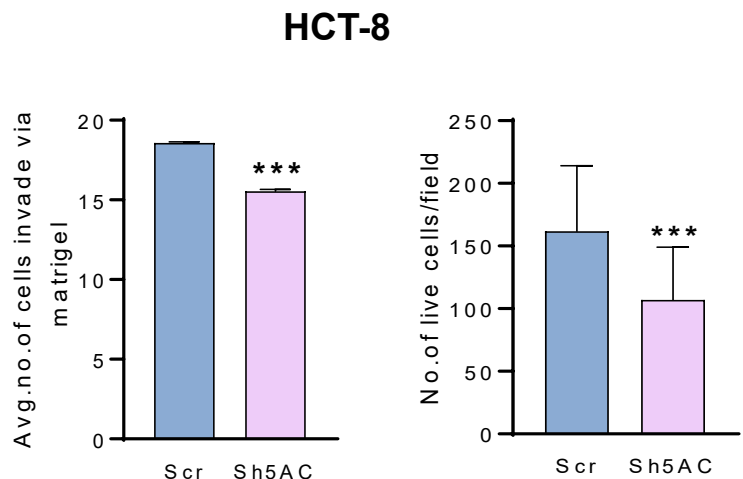

**C**

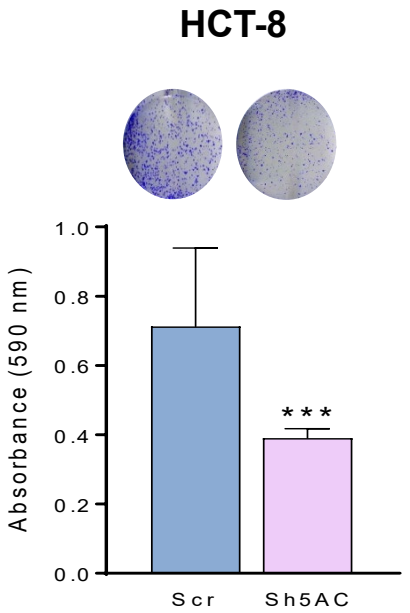

**LS174T**

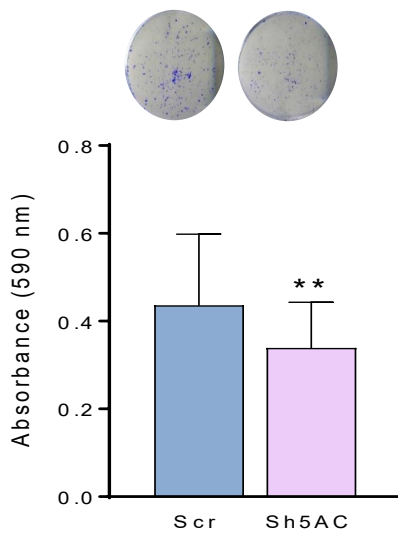

**E**

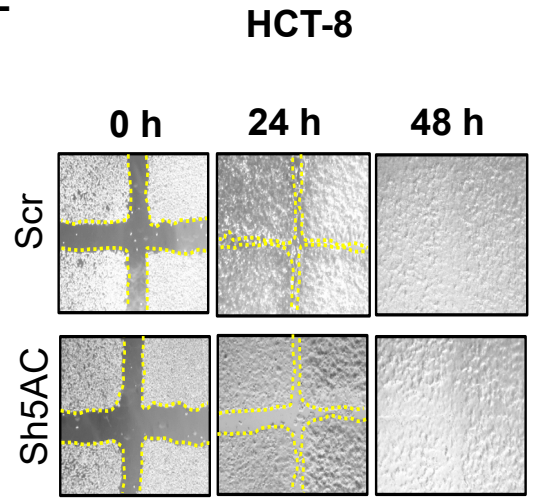

Supplementary-2

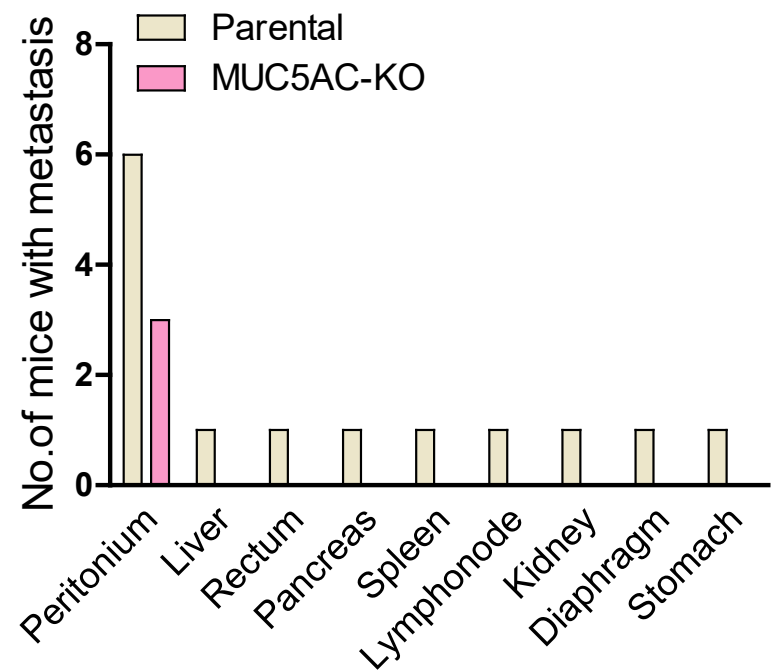

HCT-8

A

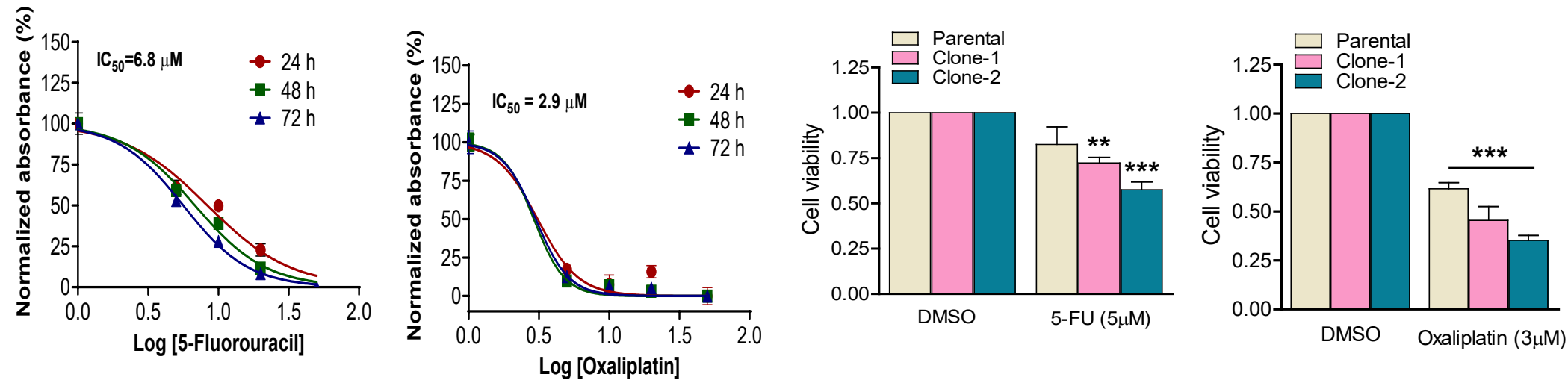

B

LS174T

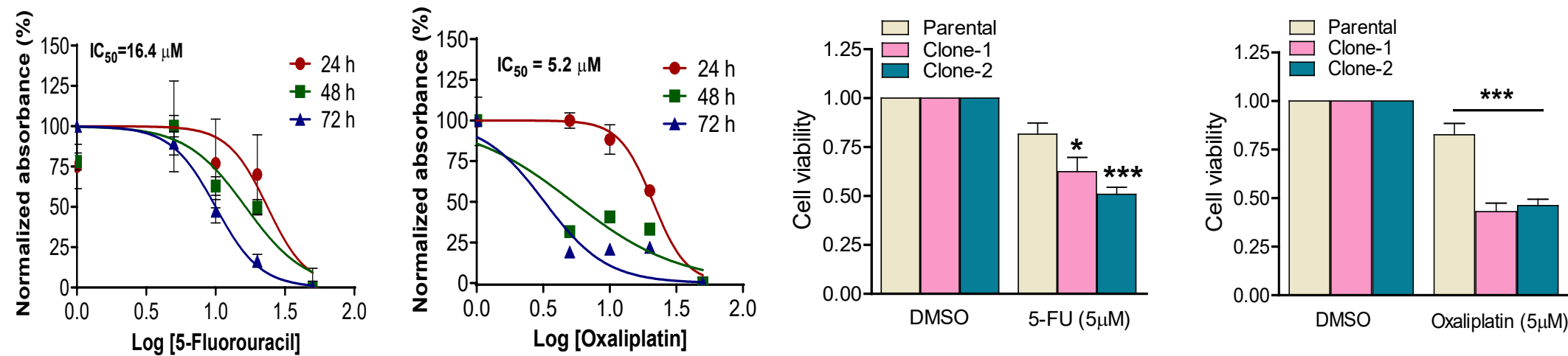

Supplement: Supplementary file 1 — Additional file 1 : Figure S1. MUC5AC mediates colon cancer cell motility, migration, and invasion. (A) MUC5AC expression was up-regulated in human CRC cell lines (HCT-8 and LS174T) as compared to a normal colon cell line (CCD 841 CoN). A549 (lung cancer cell line) was used as a positive control. (B) Cell viability of HCT-8 and LS174T cell lines stably transfected with Scr and Sh5AC was assessed by MTT assay. (C) Colony formation assay showed fewer colonies in Sh5AC cell lines compared with Scr. (D) The presence of MUC5AC in Scr cell lines increased colon cancer migration (by Boyden chamber) and invasion (by matrigel coating). (E) Sh5AC cells exhibited less cell migration than Scr cells in the wound healing assay. Figure S2. Animals with orthotopic implantation of parental MUC5AC cell lines showed different metastatic lesions as compared to knockout group. Figure S3. MUC5AC knockout sensitizes colon cancer cells to 5-FU treatment. (A and B) Cell viability was measured by MTT assay. Bar diagram indicating 5-FU and oxaliplatin treatment for 48 h significantly decreased cell viability in KO clones (Clone-1 and -2) compared with parental HCT-8 and LS174T CRC cell lines. [file 12943_2020_1156_MOESM1_ESM.pdf]
